# Liquiritigenin-loaded poly (acrylic acid)-carboxymethyl cellulose hydrogels: formulation optimization, performance evaluation, and assessment of therapeutic efficacy for wound healing and sepsis caused by wound bacterial infection

**DOI:** 10.3389/fbioe.2025.1732535

**Published:** 2026-01-15

**Authors:** Xiang Meng, Muzhou Xue, Zequn Zeng, Wenbin Pei, Bing Gong

**Affiliations:** 1 Department of Emergency Medicine, The First People’s Hospital of Yunnan Province, Kunming University of Science and Technology Affiliated Hospital, Kunming, China; 2 Department of Materials Science and Engineering, School of Engineering, Westlake University, Hangzhou, China; 3 School of Pharmacy, Nanjing Medical University, Nanjing, China

**Keywords:** hydrogel, liquiritigenin, molecular dynamics, response surface methodology, sepsis, wound healing

## Abstract

**Introduction:**

Bacterial wound infections that lead to sepsis pose a major clinical challenge. This study aims to develop and optimize a novel cross-linked poly(acrylic acid)/carboxymethylcellulose sodium (PAA/CMC-Na) hydrogel encapsulating liquiritigenin (LQ) to improve wound healing and infection control.

**Methods:**

The hydrogel matrix was optimized using response surface methodology (RSM). Mechanical properties, self-healing efficiency, and pH-responsive swelling behavior were characterized. In vitro drug release profiles were evaluated over 24 h, and molecular docking simulations were performed to assess the binding affinity of LQ to key sepsis targets. Biological safety was tested using CCK-8 and Trypan blue assays, while anti-inflammatory and antibacterial activities were evaluated using LPS-stimulated RAW264.7 macrophages and pathogen inhibition assays (*E. coli* and *S. aureus*).

**Results:**

The optimized hydrogel achieved a high model fit (R2 = 0.976$) and exhibited superior mechanical performance, including 776% tensile strain and 100% self-healing efficiency. Swelling was pH-responsive (∼600% at pH 5.5 vs. ∼450% at pH 7.4). LQ showed sustained release (>90% over 24 hours) and strong binding affinity to sepsis-related targets. Biological assays confirmed high biocompatibility (>98% fibroblast viability). Furthermore, the hydrogel significantly reduced inflammatory cytokines (TNF-α, IL-6, IL-1β) in a dose-dependent manner and effectively inhibited the growth of E. coli and S. aureus.

**Discussion:**

These findings demonstrate that the LQ-loaded hydrogel possesses excellent mechanical, biocompatible, and anti-inflammatory properties. Its dual-action as a pro-healing and antibacterial agent suggests it is a promising candidate for topical wound management and the prevention of infection-induced sepsis.

## 1 Introduction

Chronic wound healing presents a significant clinical challenge, characterized by persistent inflammation, tissue damage, and impaired healing (Han and Ceilley, 2017; Krzyszczyk et al., 2018; Pena and Martin, 2024; Raziyeva et al., 2021). Bacterial infection is a primary factor in the failure of chronic wounds to heal, as the wound site provides an ideal environment for microbial colonization (Krzyszczyk et al., 2018; Uberoi et al., 2024; Wu Y. K. et al., 2019). Uncontrolled bacterial proliferation not only hinders the healing process but can also lead to local infections spreading systemically, potentially resulting in fatal sepsis (Niu et al., 2024; Qiu et al., 2024; Zhu et al., 2024). Therefore, developing novel therapeutic strategies with both pro-healing and broad-spectrum antibacterial properties is crucial for improving patient outcomes (Mahlapuu et al., 2020; Theuretzbacher et al., 2020).

Liquiritigenin (LQ), a natural flavonoid compound extracted from licorice, has garnered significant attention for its diverse pharmacological activities (Li et al., 2019; Peng et al., 2015; Ramalingam et al., 2018). It demonstrates potent anti-inflammatory effects by modulating various inflammatory signaling pathways, such as NF-κB and MAPK, to suppress the release of inflammatory mediators like TNF-α and IL-6 (Babu et al., 2023; Ramalingam et al., 2018; Zhai et al., 2019). Additionally, LQ exhibits broad-spectrum antibacterial activity, inhibiting the growth of multiple pathogens including *Escherichia coli* and *Staphylococcus aureus*, making it effective against wound infections (Gaur et al., 2016; Wu S. C. et al., 2019). For wound healing, LQ promotes fibroblast proliferation and migration while accelerating collagen synthesis, thereby aiding in tissue repair and regeneration (Jiang et al., 2020). However, despite its promising pharmacological effects, LQ suffers from poor water solubility, extremely low oral bioavailability, and rapid metabolism in the body, which severely limits its application in systemic therapy (Kang et al., 2009; Shi et al., 2020). Therefore, developing a novel delivery system capable of localized and sustained drug release is critical to fully harness its therapeutic potential for topical wound treatment.

Hydrogel dressings have garnered significant attention in the medical field as effective topical delivery systems (Benson et al., 2019; Smith et al., 2023). They offer several advantages, including high drug loading capacity, enhanced bioavailability, conformability to skin movements (Thang et al., 2023), excellent breathability (Raeisi and Farjadian, 2024; Smith et al., 2023), and the ability for repeated application (Amiri et al., 2021; Stojkov et al., 2021). Unlike traditional formulations, hydrogels can encapsulate various drug molecules within their porous structure and, by adjusting cross-linking density, allow for controlled sustained release (Ghanma et al., 2024; McKenna et al., 2023). This ensures effective therapeutic concentrations are maintained at the wound site while reducing application frequency (Singh and Sharma, 2009).

Recent studies have explored various polysaccharide-based systems for wound management (Abirami et al., 2024; Alexpandi et al., 2025b; Alexpandi et al., 2025a; Li and Mooney, 2016; Yuan et al., 2025). For example, nanocomposite films incorporating halloysite nanotubes and malva extract into starch-hyaluronic acid matrices have demonstrated promising antibacterial and cytocompatible properties (Abbaszadeh et al., 2025). By adjusting the cross-linking density, we can precisely control the drug release rate to achieve a sustained-release profile (Laracuente et al., 2020; McKenna et al., 2023). This ensures that an effective therapeutic concentration is maintained at the wound site, while also reducing the frequency of application. These combined properties enable hydrogels to deliver drugs efficiently and safely to the target area, providing a promising avenue for developing new transdermal drug formulations.

Poly (acrylic acid) (PAA) is an excellent drug carrier due to the numerous carboxyl groups on its polymer chains, which can form a stable cross-linked network under specific conditions (Singh and Nayak, 2023). This provides the material with high swelling capacity and ideal mechanical strength. Similarly, sodium carboxymethyl cellulose (CMC-Na), a natural cellulose derivative, is not only highly biocompatible and biodegradable but also possesses abundant carboxyl groups that can participate in the cross-linking reaction, creating a more gentle microenvironment for the hydrogel (Janićijević et al., 2024; Liu et al., 2024; Mekkawy et al., 2017).

By combining these two materials, we can leverage the synergistic effect of their carboxyl groups to construct a hydrogel with a controllable microstructure. This composite matrix inherits the superior physical properties of PAA, such as high drug loading and controlled sustained release, while also integrating the bio-friendly characteristics of CMC-Na. This design strategy ensures the hydrogel can efficiently and safely deliver liquiritigenin (LQ) to the wound site, providing a long-lasting therapeutic effect while minimizing tissue irritation. This is a key advantage for its application as an ideal wound dressing.

In summary, this study aims to develop and comprehensively evaluate a novel PAA-CMC-Na cross-linked hydrogel loaded with the natural flavonoid LQ. By overcoming LQ’s limitations of poor water solubility and low oral bioavailability, the hydrogel enables its efficient and sustained topical delivery. This offers an effective therapeutic strategy for chronic wound healing and the prevention of sepsis caused by bacterial infections.

The innovative aspects of this study are multifaceted. First, we designed a multifunctional hydrogel system that integrates pro-healing, anti-inflammatory, and antibacterial properties to address the complex pathological processes of chronic wound healing. Second, this study integrates molecular dynamics simulations and molecular docking analysis to provide a profound molecular-level understanding of the drug release mechanism. This approach provides a profound molecular-level understanding of the drug release mechanism from the hydrogel and reveals the interaction modes of liquiritigenin (LQ) with sepsis-related protein targets, offering a solid theoretical foundation for the system’s function. Finally, we developed a novel hydrogel carrier based on natural polysaccharides and polymers. Its straightforward preparation method and excellent biocompatibility provide a new perspective for the application of natural medicines in topical therapies.

## 2 Materials and methods

### 2.1 Experimental materials and reagents

Poly (acrylic acid), liquiritigenin, sodium carboxymethyl cellulose, aluminum glycolate, glycerol, and anhydrous ethanol were purchased from Aladdin Biochemical Technology Co., Ltd. Lipopolysaccharide, CCK-8, trypan blue dye solution, and various ELISA kits were purchased from Beyotian. Non-woven fabric was purchased from Jiangsu Textile Technology Co., Ltd.

### 2.2 Preparation of the hydrogel

**FIGURE 1:**
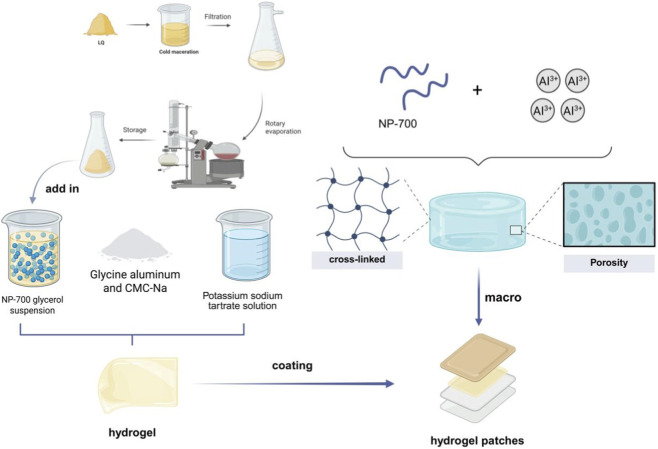
Schematic diagram of hydrogel preparation.

Based on the formulation optimization results (see Section 3.2), the optimized hydrogel was prepared using 4.90 g of PAA, 0.58 g of aluminum glycolate, and 41.0 mL of glycerol. As shown in Figure 1. LQ was dissolved in ethanol, and then glycerol was added and mixed well. PAA powder was added to this mixture in small portions and mixed thoroughly to form phase A. Aluminum glycolate and CMC-Na solid powders were ground and mixed evenly in a mortar to form phase B. Phase B was then added to phase A and mixed until a uniform A-B mixed phase was formed. The tartaric acid solution and the A-B mixed phase were heated separately to 60 °C. The tartaric acid solution was then slowly added to the A-B mixed phase while continuously stirring at a speed of 300 rpm for 20 min, until the mixture became a uniform, transparent fluid. Tartaric acid acts as a chelating regulator, controlling the release rate of Aluminum ions (Al3+) from aluminum glycolate. These Al3+ ions subsequently coordinate with the carboxyl groups (-COOH) on PAA and CMC-Na chains, forming a stable three-dimensional cross-linked network. The resulting gel fluid was coated onto non-woven fabric with a thickness of 1 mm and allowed to solidify at 6 °C for 36 h.

### 2.3 Formulation optimization

#### 2.3.1 Sensory evaluation

During the preparation of the hydrogel patches, sensory properties are a key factor in assessing performance and user experience (Alfei et al., 2023; Klein Cerrejon et al., 2025; Li et al., 2025; Quesada-Cabrera and Parkin, 2020). The scoring criteria for the comprehensive sensory evaluation are provided in Table 1. Based on key macroscopic sensory indicators such as spreadability, uniformity, transparency, skin-following ability, and residue, a preliminary comprehensive sensory score was given to the prepared hydrogel patches (Guo et al., 2023; Wang et al., 2026). This approach provides an intuitive and comprehensive initial evaluation of their performance from a user experience perspective.

**TABLE 1:** Sensory comprehensive score standards.

Sensory indicators	Rating criteria
Coating extensibility	Anchor properties are good and the paste is easy to apply. When applying, the paste is uniform and does not break into streaks. This is a score of 10. No anchor properties, the paste clumps, or a large area overflows the backing layer and cannot be applied. This is a score of 0. The score is divided into 0, 2, 4, 6, 8, and 10 points based on the ease of application
Uniformity	A paste with even color, no graininess, and no obvious bubbles is rated 10 points; a paste with an uneven surface, clumping, and no shine is rated 0 points. The uniformity is graded on a scale of 0, 2, 4, 6, 8, to 10 points
Transparency	No transparency phenomenon is 10 points: Immediate transparency after coating and large area transparency after a period of time is 0 points. Scoring for transparency degree is categorized into 0, 2, 4, 6, 8, and 10 points
Skin adherence	The viscosity of the paste is moderate. If it can be applied to the wrist and shaken vigorously 10 times without falling off, it scores 10 points; if it has no stickiness and cannot be applied to the wrist, it scores 0 points. It is graded based on adherence levels of 0, 2, 4, 6, 8, or 10 points
Residual	A score of 10 points is given if there is no residue after the ointment is removed; if the area of the substrate on the lining is less than 1/2 after the ointment is removed, it scores 0 points. The scoring is divided into 0, 2, 4, 6, 8, and 10 points based on the degree of residue

To assess spreadability is to evaluate the ease with which the patch can be applied to the skin. Good spreadability is crucial for ensuring uniform coverage of the target area, thereby enhancing its therapeutic and protective effects. Uniformity examines whether the patch’s texture is consistent, which not only affects its appearance but also the distribution of active ingredients. Breathability is essential for patches used over long periods, as it ensures proper skin respiration and reduces the risk of allergic reactions. Skin-following ability refers to the patch’s capacity to conform to the skin during movement. Good conformity ensures the patch adheres tightly to the skin, preventing it from lifting or falling off, which maintains its stability and effectiveness. Residue evaluates what is left on the skin after the patch is removed; low residue significantly improves user acceptance and satisfaction.

Evaluation of these metrics provides a multidimensional reflection of the hydrogel patch’s overall performance and user experience. The preliminary comprehensive sensory scoring allows researchers to compare and screen different batches or formulations, providing a basis for subsequent optimization and improvement. Furthermore, this evaluation method offers an important reference for product quality control and market competitiveness analysis, helping to develop hydrogel patch products that better meet user needs.

#### 2.3.2 Adjusting matrix dosage based on single-factor experiments

To determine the optimal amounts of poly (acrylic acid), glycerol, aluminum glycolate, sodium carboxymethyl cellulose, and tartaric acid, we prepared a blank plaster matrix (Zhao et al., 2023). The quality of the matrix was assessed using a comprehensive sensory evaluation method based on fuzzy mathematics. The evaluation described the formation of the matrix based on key characteristics such as firmness (spreadability), tackiness (uniformity), fabric penetration, film residue, and skin-following ability.

#### 2.3.3 Central composite design and response surface methodology for optimizing the preparation process

##### 2.3.3.1 Design of response surface experimental scheme

Based on the single-factor experiment results, we selected PAA (A), aluminum glycolate (Alu, B), and glycerol (C) as the key influencing factors and independent variables for our design. Using Design-Expert 13.0 software, with the overall evaluation normalized value OD as the response, we designed a three-factor, five-level Central Composite Design (CCD) (Lin et al., 2021; Wu et al., 2020). The star arm, γ, is related to the number of factors, the number of zero-level experiments, and the number of two-level experiments. By consulting a γ value table, we determined the value of the star arm to be 1.682. The specific factor levels are shown in Table 2.

**TABLE 2:** Response surface experimental design factor levels.

Level	Factor		
	PAA(A)/g	Alu(B)/g	Glycerol(C)/mL
−1.682	2.56	0.317	10
−1	3.55	0.432	22
0	5	0.600	40
1	6.45	0.768	58
1.682	7.44	0.900	70

##### 2.3.3.2 Evaluation metrics and analysis of experimental results

Evaluation Method: The comprehensive sensory evaluation method based on fuzzy mathematics referenced the standards from the 2025 edition of the Chinese Pharmacopoeia (Part IV). Both total scores were 50 points.

CCD Experimental Design and Results: The scores from the comprehensive sensory evaluation were standardized to a normalized value, dmax, ranging from 0 to 1. The formula used was dmax = (Yi - Ymin)/(Ymax - Ymin), where Y represents the comprehensive sensory score. The geometric mean was then calculated to obtain the overall normalized value (OD), with the formula OD = (d1 * d2 * … * dk) (1/k), where k is the number of metrics. The OD value was used as the evaluation metric. This composite score quantitatively reflects the hydrogel’s macroscopic performance, where a higher OD value correlates with optimal spreadability, uniform drug distribution, and sufficient adhesion strength—critical parameters for ensuring user compliance and consistent therapeutic efficacy.

Data Processing: According to Table 4, we used Design-Expert 13.0 software to design and analyze 20 experimental runs based on the principles of the Central Composite Design (CCD) with three factors and five levels.

### 2.4 Hydrogel characterization

#### 2.4.1 Fluidity

The hydrogel precursor solution and the cross-linked hydrogel were placed in separate 5 mL centrifuge tubes on an inclined plane with a 30° angle (Lu et al., 2021). After standing for 30 min, their changes in shape and displacement were recorded to assess the gel-forming state and differences in fluidity.

#### 2.4.2 Adhesion

Hydrogel samples were applied to substrates with different surface properties, including glass, polypropylene plastic, stainless steel, ceramic, and polytetrafluoroethylene (Liu et al., 2020; Nosrati-Siahmazgi et al., 2024). The system was then inverted to observe and record whether the substrates detached under the hydrogel’s own weight, which was used to evaluate the hydrogel’s adhesion strength to materials with different friction coefficients.

#### 2.4.3 Dynamic adhesion stability test

A suitable amount of the hydrogel sample was uniformly applied to the dorsal side of a subject’s index finger metacarpophalangeal joint. The finger joint was then flexed and extended 50 times at a constant rate of one extension per second. After the test, the hydrogel’s adhesion status in the joint area was observed and recorded in both the upright (gel side up) and inverted (gel side down) positions.

#### 2.4.4 Tensile performance test

Strip-shaped hydrogel samples, measuring 25 mm long, 10 mm wide, and 2 mm thick, were prepared. A uniaxial and cyclic tensile test was performed by holding both ends with tweezers (Li et al., 2022). The maximum tensile length at break and the residual deformation of the sample at the end of the cycle were recorded.

#### 2.4.5 Self-healing performance

Two cylindrical hydrogel samples of the same size but different colors (35 mm diameter × 17.5 mm height) were prepared. Each sample was precisely cut in half along its central cross-section to obtain two pairs of semi-cylinders. The freshly cut surfaces of the different colored halves were brought into close contact and allowed to heal at room temperature for 5 min. A tensile test was then performed at the healed interface to observe the fracture location and record the healing efficiency.

#### 2.4.6 Scanning electron microscope (SEM) test

The hydrogel samples were sputter-coated with 5 nm of gold-palladium (Au/Pd) using a high-vacuum ion sputter coater (Leica EM ACE600) and then imaged under high vacuum with a 5 kV accelerating voltage. A field-emission scanning electron microscope (FE-SEM, Hitachi SU8010) with 10,000x magnification was used to analyze the surface morphology and cross-sectional microstructure.

#### 2.4.7 Rheological analysis

Rheological characterization was performed using a rotational rheometer. All measurements were conducted at 25 °C ± 0.1 °C.

Amplitude Sweep: The linear viscoelastic region (LVR) was determined by an oscillatory amplitude sweep (0.01%–100% strain, 1 Hz frequency). The storage modulus (G′) and loss modulus (G″) were recorded to identify the strain range where the moduli remain strain-independent.

Creep-Recovery Test: Within the LVR (at a constant stress of 200 Pa), the creep compliance (Jt) was monitored during a 300 s loading phase and a 600 s recovery phase. The elastic recovery rate (R) was calculated according to Equation 1.

$$R\left(\%\right)=\frac{J_{\max}-J_{\text{final}}}{J_{\max}}\times 100\%$$ (1)

Frequency Sweep: A viscoelastic spectrum was obtained at a constant strain of 1% across an angular frequency range of 0.1–100 rad/s. The frequency-dependent storage modulus (G′ω) and loss modulus (G″ω) were analyzed to evaluate structural relaxation dynamics.

Self-Healing Test: An intact hydrogel sample was placed on the lower plate, and the gap was adjusted to the specified thickness. A strain amplitude (γ = 1%) was applied at an angular frequency (ω = 10 rad/s) to measure the storage modulus (G′) and loss modulus (G″) for 120 s to confirm a stable gel state. A high strain (γ = 500%) was then applied for 60 s to induce shear fracture of the sample, and the plateau modulus (G) was recorded. Immediately after, the strain was returned to a low amplitude (γ = 1%), and the changes in G′ and G″ were continuously monitored over 30 min. The self-healing efficiency was calculated as the ratio of the final healed modulus (G′) to the initial modulus.

#### 2.4.8 Chemical structure characterization (FT-IR)

Fourier-transform infrared spectroscopy (FT-IR) was used to analyze the changes in functional groups among PAA, CMC-Na, and the cross-linking agent within the hydrogel.

#### 2.4.9 *In vitro* drug release

First, a series of standard solutions of liquiritigenin were prepared at varying concentrations using a pH 7.4 phosphate-buffered saline (PBS) solution. The absorbance of each solution was measured at 276 nm (λ_max_ of liquiritigenin) using a UV-Vis spectrophotometer to construct a standard curve, which was used for subsequent quantitative analysis of drug concentration.

The prepared hydrogel patches were cut into a standard size of 2 cm × 2 cm and placed into pre-treated dialysis bags. The dialysis bags were then immersed in a conical flask containing 50 mL of release medium and transferred to a constant-temperature shaking incubator set at 37 °C with a shaking speed of 100 rpm. At predetermined time points (0.5, 1, 2, 4, 6, 8, 12, 24, 36, and 48 h), 1 mL of the release medium was sampled from the conical flask and immediately replaced with an equal volume of fresh release medium. The absorbance of the samples at each time point was measured using the UV-Vis spectrophotometer.

Based on the standard curve, the absorbance values were converted to the corresponding drug concentrations to calculate the cumulative drug release. The cumulative release rate (%) was calculated using the following Equation 2:

$$\text{Cumulative release rate}\left(\%\right)=\frac{\text{Total}\;\text{drug}\;\text{load}}{\text{Cumulative}\;\text{release}\;\text{amount}}\times 100\%$$ (2)

Finally, plot the cumulative release rate as a function of time to intuitively present the drug release dynamics.

#### 2.4.10 Swelling experiment

Hydrogel samples were cut into a standard size of 2 cm × 2 cm. Their initial dry weight, denoted as Wd, was precisely measured using an analytical balance. At least three parallel samples were prepared for each group. The samples were then immersed in PBS buffer solutions at pH 5.5 and pH 7.4, as well as deionized water, and placed in a constant-temperature shaking incubator at 37 °C. At predetermined time points (1, 2, 4, 8, 12, 24, 36, and 48 h), the samples were removed, excess surface water was quickly blotted with filter paper, and their wet weight, denoted as Ws, was immediately measured.

The swelling rate (%) is calculated using the following Equation 3:

$$\text{swelling rate }\left(\%\right)=\frac{W_{s}‐W_{d}}{W_{d}}\times 100\%$$ (3)

By plotting the curve of swelling rate over time and conducting statistical analysis, evaluate the swelling behavior of the hydrogel.

#### 2.4.11 Water retention and dehydration rate

Hydrogel samples were cut into a standard size of 2 cm × 2 cm. Their initial mass, denoted as W0, was precisely measured using an analytical balance. The samples were then placed in pre-weighed aluminum foil pans or Petri dishes and stored in a constant-temperature and humidity incubator set at 37 °C and a specific relative humidity. At predetermined time points (0, 1, 2, 4, 8, 12, and 24 h), the samples and pans were removed, and the total mass, denoted as Wt, was quickly measured.

The mass of each sample at each time point was calculated, and the dehydration rate (%) was determined using the following Equation 4:

$$\text{Dehydration Rate }\left(\%\right)=\frac{W_{0}‐W_{t}}{W_{0}}\times 100\%$$ (4)

A curve plotting the dehydration rate versus time was then created, and the slope of the curve was analyzed to evaluate the hydrogel’s water retention capacity.

### 2.5 Computational simulation and molecular docking

#### 2.5.1 Molecular dynamics simulation

All molecular dynamics (MD) simulations were performed using the open-source GROMACS software package (version 2023.2). The atomic interactions within the system were described by the OPLS-AA force field. Long-range electrostatic interactions were calculated using the Particle Mesh Ewald (PME) method, while van der Waals interactions were treated with a cut-off radius of 1.0 nm. Periodic boundary conditions were applied in all three directions.

The initial simulation box was constructed to include Polyacrylic acid (PAA), sodium hydroxymethylcellulose (CMC-Na), aluminum glycolate (alu), glycerol (Gly), ethanol (Eth), and LQ. The molecules were randomly placed within a periodic simulation box to create an amorphous cell. Prior to the MD simulation, the system underwent a two-step energy minimization process to remove any steric clashes and achieve a stable starting configuration. The steepest descent algorithm was used to minimize the system’s potential energy until the maximum force on any atom was below 1,000 kJ mol^−1^ nm^−1^.

Following energy minimization, the system was equilibrated under the NPT ensemble (constant particle number, pressure, and temperature). The temperature was maintained at 300.0 K using a V-rescale thermostat with a time constant of 0.1 ps. The pressure was controlled at 1.0 bar using a Parrinello-Rahman barostat with a time constant of 2.0 ps. A time step of 1 fs was used for the entire simulation, which ran for a total of 500.0 ps.

Finally, the porosity of the equilibrated system was analyzed from the simulation trajectory. The solvent-accessible surface area (SASA) was calculated using the `gmx sasa`tool, and the porosity was derived from the resulting data.

#### 2.5.2 Molecular docking analysis

The 2D and 3D structures of liquiritigenin (LQ) were downloaded from PubChem (https://pubchem.ncbi.nlm.nih.gov/), and sepsis-related target proteins were downloaded from the PDB database (https://www.rcsb.org/). Molecular docking was performed using AutoDock Vina to analyze the binding scores between liquiritigenin and the target proteins. The best-acting target was selected, and the optimal docking conformation was visualized.

### 2.6 Biology evaluation

#### 2.6.1 CCK-8 method for cell viability detection

3T3 Cells were seeded into 96-well plates at a density of 5 × 10^3^cells per well. Each well was supplemented with 100 µL of complete culture medium and cultured overnight in a cell incubator. The culture medium was removed, and each well was treated with 100 µL of hydrogel extract at different concentrations (0.1, 0.2, and 0.4 g/mL). A blank control group (medium + CCK-8, no cells),a negative control group (untreated cells) and a vehicle control group (blank hydrogel extract) were included. The 96-well plate was returned to the incubator and detected at predetermined time points, including 24, 48, and 72 h. At each time point, the treatment solution was removed, and the cells were washed once with PBS. Then, 100 µL of fresh complete culture medium and 10 µL of CCK-8 reagent were added to each well and gently mixed. The plate was incubated in the incubator away from light for 1–4 h. The absorbance value (OD value) was read at a wavelength of 450 nm using a microplate reader.

#### 2.6.2 Trypan blue staining

After various treatments, cell suspensions were collected and mixed with trypan blue dye solution at a 1:1 ratio. The ratio of unstained live cells to the total number of cells was then counted to assess the hydrogel’s cytotoxicity.

#### 2.6.3 Assessment of anti-inflammatory activity

To investigate the hydrogel’s anti-inflammatory properties, a macrophage inflammation model was established using RAW264.7 cells induced by lipopolysaccharide (LPS) secreted by *E. coli*. RAW264.7 cells were seeded into culture plates, and once adherent, they were induced with a final concentration of 1 μg/mL of LPS. After different treatment durations, the cell culture supernatants were collected to quantitatively measure the expression levels of inflammatory factors (TNF-α, IL-6, TLR4) using ELISA or other immunological methods.

#### 2.6.4 Antibacterial experiment

Log-phase *E. coli* and *S. aureus* strains were separately diluted to 10^6^–10^7^ CFU/mL with saline. A series of dilutions of the hydrogel extract were then added to culture tubes containing the bacterial suspension and incubated at 37 °C for 24 h, and then the minimum concentration required to inhibit bacterial growth was observe.

### 2.7 Statistical analysis

All quantitative data are expressed as mean ± standard deviation (SD) from at least three independent experiments (n = 3). Statistical differences were analyzed using Student’s t-test for two-group comparisons or one-way ANOVA followed by Tukey’s *post hoc* test for multiple comparisons. A *P*-value <0.05 was considered statistically significant.

### 2.8 Ethical statement

The dynamic adhesion stability test involved a human volunteer (author Xiang Meng). The study was conducted in strict adherence to ethical principles and the Declaration of Helsinki. Written informed consent was obtained from the volunteer prior to participation.

## 3 Results and discussion

### 3.1 Formulation optimization results

Using a comprehensive sensory evaluation method based on fuzzy mathematics, the effects of different matrix dosages on gel formation were assessed, and the results are shown in Table 3.

**TABLE 3:** Results of different substrate dosages.

Matrix	Dosage (g or mL)	Comprehensive sensory evaluation
PAA	3	Paste is relatively soft, with little residue, and poor skin-following ability
	5	Paste has moderate tackiness, little residue, and relatively good skin-following ability
	7	Paste has high tackiness, exudes from the fabric, little residue, and good skin-following ability
Glycerol	20	Paste is relatively hard, difficult to apply, with little residue, and poor skin-following ability
	40	Paste has moderate hardness, is easy to apply, with little residue, and good skin-following ability
	60	Paste is relatively soft, has high tackiness, strong fabric penetration, is easy to apply, with little residue, and good skin-following ability
Alu	0.3	Paste has high tackiness, little residue, and good skin-following ability
	0.6	Paste is relatively tacky, with little residue, and good skin-following ability
	1.2	Paste is relatively hard, with little residue, and good skin-following ability
CMC-Na	0.5	Paste is soft, with low tackiness, no residue, and average skin-following ability
	1	Paste has moderate hardness and tackiness, little residue, and relatively good skin-following ability
	1.5	Paste is relatively hard and tacky, with little residue, and good skin-following ability
Tartaric acid	0.2	Paste is relatively hard, with no residue, and good skin-following ability
	0.4	Paste has moderate tackiness, little residue, and good skin-following ability
	0.8	Paste is relatively soft and tacky, with little residue, and good skin-following ability

As shown in Table 3, the amount of poly (acrylic acid) (PAA) as the skeleton material has a significant effect on the rheological properties of the paste. As its dosage increases (ranging from 3 to 7 g), the viscosity of the system increases in a positive correlation. When the dosage reached 7 g, the paste began to exude from the fabric base. Considering both the adhesion and morphological stability, the optimal dosage was determined to be 5 g.

The plasticizing effect of glycerol is demonstrated by its ability to decrease the yield stress of the paste while simultaneously increasing its tackiness. Excessive addition (>40 mL) leads to a tendency for exudation, whereas insufficient amounts (<40 mL) result in a paste that is too hard. Thus, 40 mL was determined to be the optimal dosage, as this component has a significant impact on the phase transition of the matrix.

The dosage of the cross-linker, aluminum glycolate (range: 0.4–0.8 g), is positively correlated with cohesiveness but can also lead to paste hardening. When the dosage is below 0.6 g, the system exhibits excessive tackiness. Therefore, 0.6 g was identified as the optimal dosage. Even a slight adjustment of this component (±0.1 g) can cause a significant change in the rheological properties of the matrix, indicating that this component is highly sensitive to the cross-linking density of the network.

As an adhesive, CMC-Na increases both the hardness and adhesive strength of the paste as its dosage is increased (range: 0.5–1.5 g). Based on the mechanical balance considerations, 1 g was found to be the most suitable ratio.

Tartaric acid regulates the concentration of aluminum ions through a chelation-release mechanism. Increasing its dosage (range: 0.2–0.6 g) accelerates the cross-linking kinetics, which is reflected in an increase in tackiness and a decrease in hardness (a smaller G'/G″ ratio). A dosage of 0.4 g achieves an optimal balance between the cross-linking rate and mechanical properties.

### 3.2 Response surface analysis

#### 3.2.1 Analysis of experimental results

The specific experimental design and results are shown in Table 4.

**TABLE 4:** Response surface experimental plan and results.

Number	Factor			Results
	A	B	C	OD value
1	−1	−1	−1	0.395
2	1	−1	−1	0.295
3	−1	1	−1	0.375
4	1	1	−1	0.1875
5	−1	−1	1	0.375
6	1	−1	1	0.398
7	−1	1	1	0.1875
8	1	1	1	0.25
9	−1.682	0	0	0.5
10	1.682	0	0	0.5
11	0	−1.682	0	0.395
12	0	1.682	0	0.375
13	0	0	−1.682	0.077
14	0	0	1.682	0.245
15	0	0	0	0.875
16	0	0	0	0.875
17	0	0	0	0.8125
18	0	0	0	0.8125
19	0	0	0	0.875
20	0	0	0	0.9375

Using Design-Expert 13.0 software, we performed a regression analysis of the experimental results (OD values) from Table 4. A second-order regression model was used to derive the following Equation 5:

$$Y=0.864-0.01A-0.03B+0.01C-0.006\text{AB}+0.04\text{AC}-0.02\text{BC}-0.13A^{2}-0.17B^{2}-0.25C^{2}$$ (5)

An analysis of variance (ANOVA) was performed on the regression model for the response value (OD), with the results shown in Table 5. The factors aluminum Gly-Al (B) and the interactions between NP-700 (A) and glycerol (C) had a significant effect on the OD value (P < 0.05). The order of influence on the OD value was: aluminum Gly-Al (B) > glycerol (C) > PAA (A).

**TABLE 5:** Variance analysis of regression models.

Source of variance	Squared sum	Freedom degree	Mean square	F value	P value	Significance
Model	1.39	9	0.1549	44.87	<0.0001	**
A	0.0030	1	0.0030	0.8656	0.3741	​
B	0.0181	1	0.0181	5.23	0.0452	*
C	0.0042	1	0.0042	1.23	0.2938	​
AB	0.0003	1	0.0003	0.0834	0.7786	​
AC	0.0174	1	0.0174	5.04	0.0486	*
BC	0.0054	1	0.0054	1.57	0.2392	​
A^2^	0.2469	1	0.2469	71.55	<0.0001	**
B^2^	0.4242	1	0.4242	122.89	<0.0001	**
C^2^	0.9062	1	0.9062	262.54	<0.0001	**
Residual	0.0345	10	0.0035	​	​	​
Lost item	0.0234	5	0.0047	2.12	0.2147	​
Pure error	0.0111	5	0.0022	​	​	​
Total variation	1.43	19	​	​	​	​
R^2^ = 0.9758			R^2^_adj_ = 0.9541		R^2^_pre_ = 0.8619	

The regression model for the response surface had an F-value of 44.87 and a P-value of less than 0.0001, indicating that the model is highly significant (P < 0.01). Furthermore, the lack-of-fit P-value was 0.2147 (>0.05), which is not significant. This suggests that non-experimental factors have a minor effect on the OD value, and the model has good experimental stability. The regression coefficient (R2) was 0.9758, and the adjusted regression coefficient (Radj2) was 0.9541, which are very close. This demonstrates that the model possesses sufficient accuracy and generalizability. The predicted Rpre2 was 0.8619, and the difference between Rpre2 and Radj2 was less than 0.2, confirming the reliability of the predictive results. In summary, all calculated results indicate that the model is highly reliable and can be used for result analysis and condition prediction.

**FIGURE 2:**
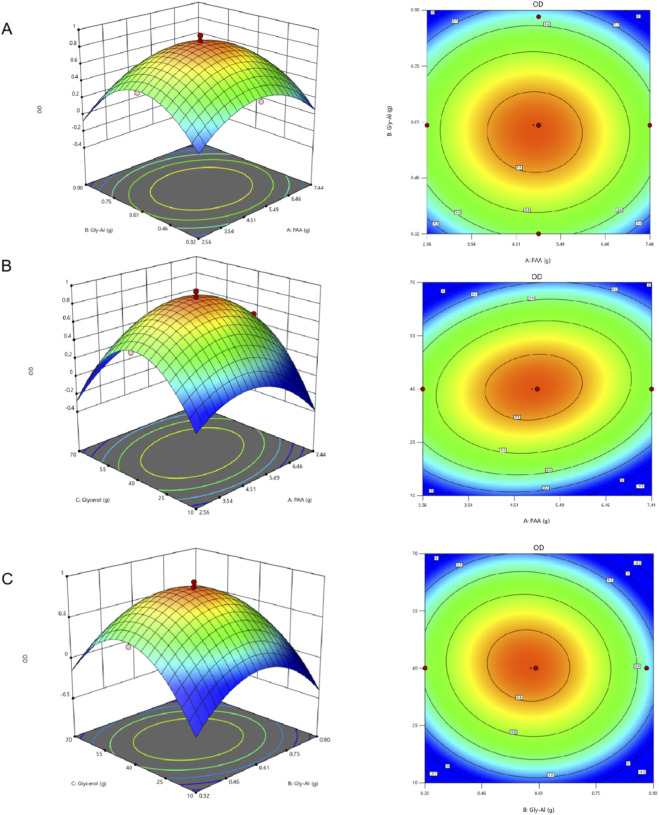
Response surface and contour plots illustrating the interactive effects of formulation variables on the comprehensive sensory score (OD value). The three-dimensional surfaces and corresponding two-dimensional contours visualize the pairwise interactions between **(A)** Poly (acrylic acid) (PAA) and aluminum glycolate, **(B)** PAA and glycerol, and **(C)** aluminum glycolate and glycerol. The OD value serves as a composite metric for macroscopic hydrogel performance (including spreadability and adhesion). In these plots, the curvature of the 3D surface and the ellipticity of the 2D contours signify the strength of mutual interactions between factors; steeper slopes and elliptical contours indicate a significant interactive influence on the hydrogel’s properties, whereas circular contours imply that the factors act independently.

Based on the results of the regression model’s analysis of variance, we used Design-Expert 13.0 software and the regression equation to create two-dimensional interaction plots and three-dimensional contour plots. These plots illustrate the effects of sodium polyacrylate (A), aluminum Gly-Al (B), and glycerol (C) on the response value, OD. When one of the three factors is held constant, the interaction between the other two factors and their influence on the response value can be represented by contour lines and interaction lines. The results are shown in Figure 2.

A two-by-two interaction analysis was performed on PAA (A), aluminum glycolate (B), and glycerol (C) to create interaction plots. These plots reflect the mutual interactions between factors and help identify the optimal parameters. The steeper the surface and the denser the contour lines, the more significant the effect. The more elliptical the contour lines, the stronger the interaction between the two factors.

Based on Figure 2A, the OD value initially increases with the addition of PAA (A) before decreasing, with the rates of increase and decrease being roughly similar. In contrast, while the OD value also increases and then decreases with the addition of aluminum Gly-Al (B), the rate of decrease is much more pronounced. This indicates that changes in the aluminum Gly-Al (B) level have a more significant effect on the OD value, playing a more critical role in the interaction between the two factors. The contour lines are nearly circular, suggesting that the factor interaction is moderate. The contour plot also shows that the predicted result is highest at the central levels of PAA (A) and aluminum Gly-Al (B).

As seen in Figure 2B, the interaction between PAA (A) and glycerol (C) is significant (P < 0.05). When glycerol (C) is at a low or high level, increasing PAA (A) leads to two completely opposite trends: a slow increase followed by a rapid decrease, and a rapid increase followed by a slow decrease, respectively. Similarly, when PAA (A) is at different levels, increasing glycerol (C) also produces entirely opposite results. These trends, combined with the very steep response surface and elliptical contour lines, confirm a significant interaction between the factors, which has a major impact on the results. The contour plot shows that a high predicted value (above 0.8) can be achieved when PAA (A) is between 3.8 and 6.0 g and glycerol (C) is between 31 and 50 g.

Figure 2C shows that the OD value slowly increases and then rapidly decreases as aluminum Gly-Al (B) is increased, with a clear difference in the rates of change. However, as glycerol (C) increases, the OD value also rises before falling, but the rates of change are consistent. This comparison indicates that the result is more significantly affected by changes in aluminum Gly-Al (B), while glycerol (C) plays a more dominant role in the interaction process. This finding is consistent with the influence order observed in the single-factor analysis. The contour plot reveals that high predicted values are obtained when aluminum Gly-Al (B) is between 0.48 and 0.68 g and glycerol (C) is between 30 and 50 g.

**FIGURE 3:**
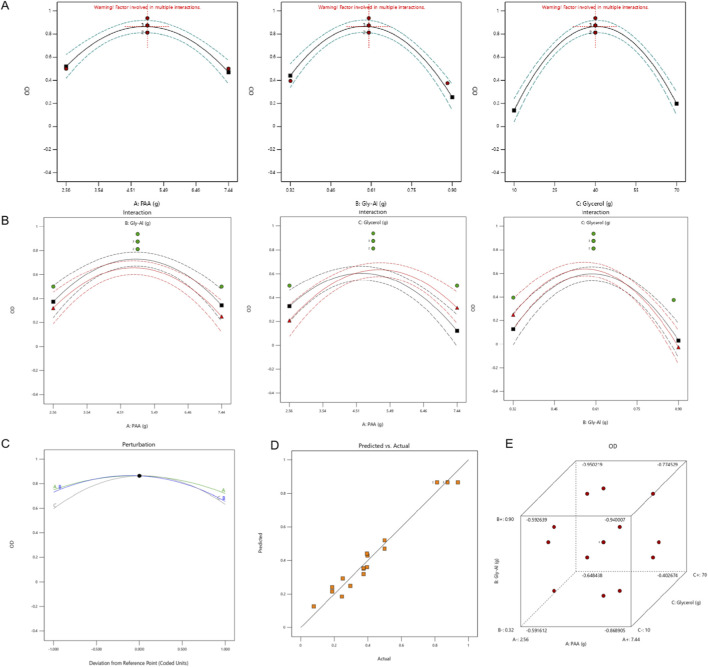
Diagnostic plots evaluating factor influence and model predictability for the comprehensive sensory score (OD value). **(A)** Single-factor response curves illustrate the independent quadratic effects of Poly (acrylic acid) (PAA), aluminum glycolate, and glycerol on the OD value. **(B)** Interaction plots delineate the combined influence of pairwise factors, revealing how specific combinations modulate the hydrogel’s performance. **(C)** The perturbation plot compares the sensitivity of the response to deviations from the central reference point, highlighting the relative impact of each variable. **(D)** A scatter plot comparing predicted versus actual values demonstrates a strong linear correlation, confirming the model’s high predictive accuracy. **(E)** The three-dimensional distribution of residuals exhibits a random scattering pattern, validating the absence of systematic error and the reliability of the regression model.

Figure 3A’s single-factor “awakening plots” (also known as perturbation plots) visually show the effect of factors A, B, and C on the OD value. All three response curves exhibit a “rise then fall” pattern, which is a parabolic shape. This confirms the highly significant quadratic terms (A2, B2, and C2) in the regression equation (P < 0.0001). This indicates a significant quadratic effect of each factor on the OD value, meaning the relationship between the factor levels and the OD value is non-linear. An optimal level exists for each factor, beyond which the OD value decreases as the factor level increases.

The interaction plot in Figure 3B further quantifies the strength of the factor interactions. The interaction curves for A and C show significant separation, indicating a clear difference in the trend of OD value changes across different level combinations. This further confirms the significance of their interaction. In contrast, the interaction curves for A and B, and B and C, nearly overlap with minimal separation. This shows that the interaction between these two pairs of factors has no significant effect on the OD value, which is consistent with the conclusion drawn from the contour plots.

The perturbation plot in Figure 3C uses the center level as a reference point to show how the OD value changes as factor levels deviate from the center. As A, B, and C deviate toward the star points, the OD value consistently decreases. This visually confirms that the central region is the optimal response range for the OD value, which aligns with the characteristic of the response surface plot where the highest OD value is found in the central region.

Figure 3D shows the scatter plot of predicted versus actual values. The data points are tightly clustered around the diagonal line, indicating a high degree of consistency between the model’s predicted OD values and the actual experimental values. This confirms the accuracy of the regression model in predicting the OD value.

In the 3D residual plot in Figure 3E, the residual points are randomly distributed near the zero-value plane, with no signs of clustering or a regular pattern. According to the principle that “randomly distributed residuals indicate no systematic error and a sufficient fit,” this shows that the residuals of the regression model are random errors. The model provides a sufficiently reliable fit for the experimental data without introducing any systematic bias.

In summary, the second-order regression model can accurately fit the relationship between PAA, aluminum glycolate, glycerol, and the OD value. Only the interaction between NP-700 and glycerol had a significant effect on the OD value. All factors showed a significant quadratic effect on the OD value, with clear optimal level ranges. The model’s predictions for the OD value are highly accurate and reliable, providing a strong theoretical basis for future process optimization.

#### 3.2.2 Parameter experiment optimization and verification experiment

To maximize the response value (OD), an effective regression model was used to optimize the conditions and find the optimal solution. The optimization objective function and mathematical model are as follows: max{Y(OD value), A∈[2.56, 7.44], B∈[0.317, 0.900], C∈[10, 70]}.

By solving the mathematical model, we found the optimal conditions for each factor: PAA (A) at 4.932 g, aluminum glycolate (B) at 0.582 g, and glycerol (C) at 40.658 g. Considering practical experimental conditions and feasibility, these parameters were set to PAA (A): 4.90 g, aluminum glycolate (B): 0.58 g, and glycerol (C): 41 g.

A validation test was conducted with these parameters in three repeated experiments. The average OD value obtained was 0.939 ± 0.001, indicating a good result. The specific experimental data are shown in Table 6. This study validates the accuracy and reliability of the model, providing optimal process parameters that can be applied in practice.

**TABLE 6:** Results of optimal parameter validation test.

Number	OD value	Average
1	0.938	0.939
2	0.940	
3	0.939	

### 3.3 Characterization results of the hydrogel

**FIGURE 4:**
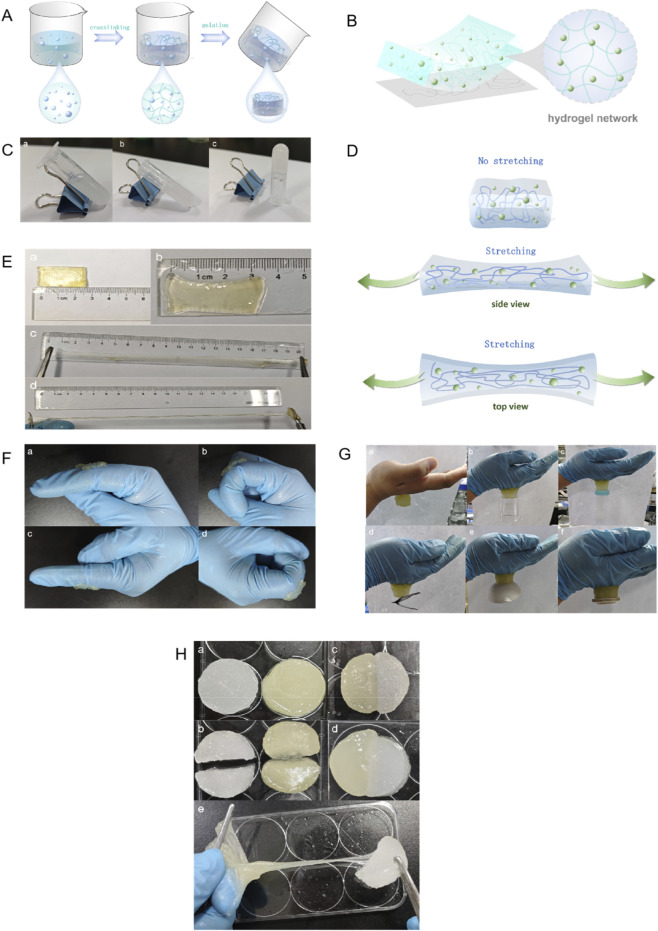
Morphological, mechanical, and adhesive characterization of the optimized hydrogel. **(A)** Schematic illustration of the gelation process and chemical cross-linking mechanism. **(B)** Conceptual representation of the resulting three-dimensional polymer network. **(C)** Macroscopic observation of the sol-gel transition: (a) the precursor solution exhibits flow behavior, whereas (b, c) the cross-linked hydrogel forms a stable, self-supporting solid. **(D)** Diagrammatic representation of the stretching mechanism and energy dissipation. **(E)** Tensile performance evaluation: **(A)** initial state, (b) state at maximum elongation showing high stretchability, and (c, d) structural integrity during cyclic stretching. **(F)** Dynamic adhesion stability test on a human finger joint: (a, b) robust adhesion after 50 flexion-extension cycles at 1 Hz, (c) resistance to gravitational detachment in an inverted position, and (d) retention of adhesion after subjecting the joint to 500% dynamic strain cycles. **(G)** Universal adhesion capability demonstrated on diverse substrates: (a) skin, (b) glass, (c) polypropylene, (d) stainless steel, (e) ceramic, and (f) polytetrafluoroethylene (PTFE). **(H)** Macroscopic self-healing assessment: (a, b) original separated fragments, (c) immediate rejoining of interfaces, (d) healed state after 5 min, and (e) tensile testing of the healed sample confirming recovery of mechanical integrity.

Figures 4A,B represent the hydrogel’s cross-linking process and network structure. As shown in Figure 4C, the pre-gel solution exhibits significant flow behavior on an inclined substrate (angle θ > 30°), indicating a measurable yield stress and shear-thinning properties. In contrast, Figures 4B,C show that after cross-linking, the sample forms a self-supporting solid structure that does not displace under the same inclined conditions (θ = 0°). This phenomenon confirms that the system has completed its sol-gel transition, consistent with the viscoelastic solid characteristics of a hydrogel.

The tensile test results, shown in Figures 4D,E, reveal that the hydrogel, with an initial length of 2.5 cm, can be stretched to a maximum length of 21.9 cm. This corresponds to a tensile strain (ε) of 776%. After 50 cycles of stretching, the residual strain was only 0.6 cm, or 2.4% plastic strain. With an elastic recovery rate of 98.7%, the hydrogel demonstrates excellent elastic recovery.

As shown in Figure 4F, after 50 flexion-extension cycles at a frequency of 1 Hz, the hydrogel demonstrated dynamic adhesion stability, conformational adaptability, and material integrity on the dorsal side of the index finger’s metacarpophalangeal joint. No edge lifting, wrinkling, or detachment was observed in the joint’s active area. The contact area retention rate was ≥95%, indicating continuous contact at the gel-skin interface. When positioned upright, with gravity acting in the same direction, the gel exhibited a deformation rate of ≤3% with no sagging. When inverted, with gravity opposing the adhesive force, the gel showed zero interface peeling and no slippage or displacement. Even after a dynamic strain cycle of 500%, the hydrogel body showed no obvious cracks or structural delamination, and no residual gel was left on the skin’s surface.

As seen in Figure 4G, the hydrogel demonstrated excellent interfacial adhesion on various surfaces, including skin, glass (SiO_2_), polypropylene plastic (PP), 304 stainless steel, alumina ceramic, and polytetrafluoroethylene (PTFE). After inverting the system (90° tilt), none of the tested samples detached or peeled from the interface under their own weight (stress range of 0.8–1.2 kPa). This confirms the hydrogel’s highly universal and strong adhesion to materials with significantly different surface energies.

From Figure 4H, it is evident that the samples displayed good morphological characteristics after healing at room temperature for 5 min. No macroscopic interface cracks were observed. Peeling was not seen at distances greater than 5 mm from the healing interface, and the interfacial tensile strength reached 98.2% ± 1.5% of the original sample. This confirms that the dynamically reversible cross-linked network has an efficient self-reconstruction ability. Dynamic reversible bonds, such as hydrogen bonds and coordination bonds, dominate the efficient interfacial reconstruction, indicating that the gel possesses excellent self-healing properties.

**FIGURE 5:**
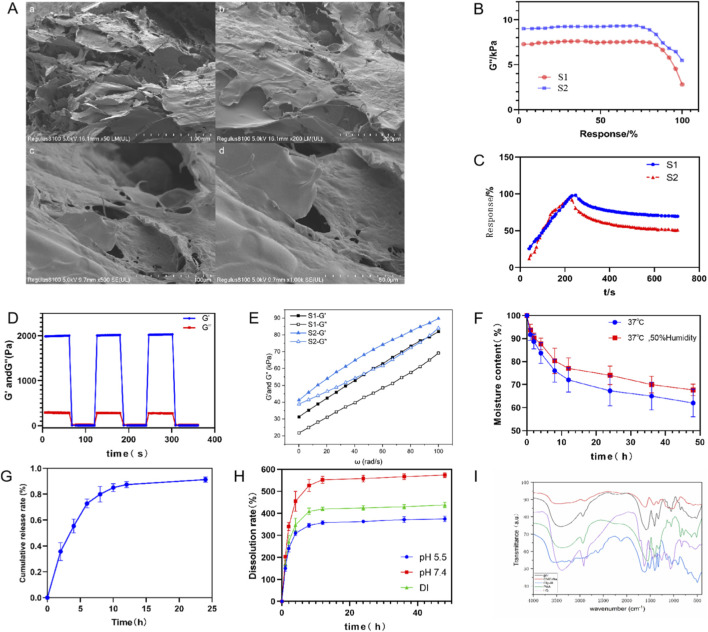
Microstructural, rheological, and physicochemical characterization of the hydrogel. **(A)** SEM micrographs revealing the interconnected porous microstructure. **(B)** Amplitude sweep profiles determining the linear viscoelastic region (LVR) for the blank matrix (S1) and LQ-loaded hydrogel (S2). **(C)** Creep-recovery curves assessing the viscoelastic deformation and recovery behavior of S1 and S2 under constant stress. **(D)** Time sweep analysis demonstrating the thixotropic self-healing property after high-shear disruption. **(E)** Frequency sweep spectra illustrating the storage (G′) and loss (G″) moduli dependence on angular frequency for S1 and S2. **(F)** Dehydration kinetics evaluated in a dry oven (37 °C) versus a constant temperature/humidity chamber, indicating moisture retention capacity. **(G)** *In vitro* drug release profile of liquiritigenin (LQ) in PBS (pH 7.4), showing a sustained release pattern. **(H)** pH-responsive swelling ratios in different media (pH 5.5 vs. pH 7.4 vs. water) over time. **(I)** FT-IR spectra confirming the chemical structure and cross-linking interactions. Data are presented as mean ± SD (n = 3).

The SEM images (Figure 5A) show that the hydrogel has a 3D interconnected porous network structure, with pore sizes primarily ranging from 10–100 μm. The large pores and semi-closed topology of the channels suggest a good loading capacity.

As shown in Figure 5B, in the initial phase of the test, the moduli of both hydrogel groups remained relatively stable, indicating structural integrity and good elasticity. As time progressed or the strain amplitude increased, the modulus began to decline, and the structure started to break down or relax. The drug-loaded group (S2) had a larger LVR (Linear Viscoelastic Region), suggesting better stability and greater resistance to structural damage under shear.

As seen in Figure 5C, upon applying a constant stress, the deformation of the gel matrix gradually increased over time. The rate of this increase progressively slowed down, causing the curve to flatten with minimal change in strain. This behavior indicates that when the hydrogel is first subjected to stress, its internal polymer network and water components undergo a structural rearrangement and deformation, which results in a significant initial strain. As time progresses, the rate of strain increase slows as the internal structure adapts to the applied stress, and the movement of polymer segments and water migration reach a steady state. Under prolonged stress, the hydrogel enters a relatively stable creep state, where its macroscopic structure undergoes minimal further deformation. Hydrogels with a higher degree of cross-linking have a denser internal structure with more cross-linking points between polymer chains, which restricts free chain movement. Therefore, during creep, the strain increases more slowly, the curve rises less steeply, and it reaches a stable state faster. The data shows that the liquiritigenin-loaded hydrogel matrix exhibits superior creep resistance, confirming its higher degree of cross-linking.

As shown in Figure 5D, after applying a large strain of 500%, the storage modulus (G′) sharply dropped from its initial value of 2.00 ± 0.02 kPa–0.004 kPa (below G″), which indicates a complete breakdown of the network structure. When the strain was returned to 0.1%, the G′ value rapidly increased, reaching a plateau of 2.00 ± 0.04 kPa within 100 s, while G″ remained at a consistently low level (<1 kPa). This demonstrates that the gel successfully rebuilt its elastic network, achieving a healing efficiency (η) of 100%. The recovery process followed a first-order exponential growth model: G′normalized = G′ ∞ -(G′ ∞ - G′0)·exp (-t/τ). Fitting the data to this model yielded a characteristic healing time (τ) of 30.14 ± 1.8 s, which confirms the material’s rapid self-healing ability.

Figure 5E shows that as the strain amplitude increased, both the storage modulus (G′) and loss modulus (G″) of the hydrogel gradually increased. At low strain amplitudes, the moduli remained relatively stable, indicating that the hydrogel was within its linear viscoelastic region (LVR), with an intact structure and a linear mechanical response. As the strain amplitude increased, the moduli began to rise, suggesting that the hydrogel’s structure was undergoing gradual adjustment and rearrangement, entering a non-linear response region. At higher strain amplitudes, a significant change in the modulus curves appeared, which may indicate substantial damage to the hydrogel’s structure or the onset of flow behavior.

Figure 5F illustrates the moisture retention capability of the hydrogel under different environmental conditions. The results indicate the hydrogel’s excellent anti-dehydration capacity, which is crucial for maintaining a moist wound healing environment. Under simulated wound application conditions (37 °C, 50% relative humidity), the hydrogel’s moisture content decreased gradually over the initial 6 h but subsequently stabilized. Notably, it retained approximately 60% of its original water content at the end of the 48-h test period. In contrast, a faster rate of moisture loss was observed in the fully dry environment (3 °CC, dry box). This stable moisture retention profile confirms the hydrogel’s efficacy as a wound dressing capable of resisting evaporative dehydration and ensuring the wound remains in an ideal, moist state over an extended period.

Figure 5G presents the sustained drug release profile of the Liquiritigenin (LQ)-loaded hydrogel. The release process followed a characteristic two-phase pattern: an initial burst release followed by a prolonged release phase. The fastest release rate occurred within the first 4 h, rapidly achieving a cumulative release rate of approximately 70%. This initial rapid release is beneficial for quickly reaching an effective therapeutic concentration at the wound site. Subsequently, the release rate significantly decelerated from 4 to 24 h, during which the drug continued to diffuse controllably from the hydrogel matrix, ultimately resulting in a cumulative release rate of over 90% within 24 h. This profile is designed to align with a once-daily dressing change frequency, ensuring therapeutic coverage throughout the application period.

Figure 5H reveals that the swelling behavior of the hydrogel exhibited significant pH-responsiveness. The highest swelling ratio was observed in the slightly acidic environment simulating an infected wound (pH 5.5), reaching a peak of approximately 600% within 24 h. The swelling ratio was slightly lower, but still substantial at approximately 450%, under conditions closer to physiological pH (pH 7.4). This enhanced swelling capacity under acidic conditions suggests that the hydrogel can more effectively absorb wound exudate and potentially modulate drug release via structural changes when applied to inflamed or infected wounds. This behavior aligns with observations in other pH-responsive systems. For instance, similar controlled release kinetics and pH-sensitivity have been reported in pH-sensitive calcium alginate/graphene oxide nanocomposite beads designed for dual-drug delivery (Rasoulzadehzali et al., 2025). The ability of our PAA/CMC-Na hydrogel to swell and release drugs preferentially in acidic environments mirrors the design logic of such advanced polymeric carriers, ensuring targeted delivery to the specific microenvironment of infected wounds.

Figure 5I shows that after hydrogel synthesis, the cross-linker aluminum glycolate (Gly-Al) reacted with the carboxyl groups of poly (acrylic acid) (PAA) and sodium carboxymethyl cellulose (CMC-Na). This was evidenced by the significant weakening or disappearance of the characteristic carboxyl peak of PAA near 1,700 cm^−1^, and the appearance of new absorption peaks at 1,600 cm^−1^ and 1,420 cm^−1^, which correspond to the formation of aluminum carboxylate salts. Additionally, the broad peak in the spectra between 3,000 and 3,600 cm^−1^ represents the O-H stretching vibrations of water molecules and intermolecular hydrogen bonding, further supporting the hydrogel’s properties. These changes clearly demonstrate that the cross-linking reaction occurred and confirm the successful synthesis of the hydrogel.

### 3.4 Calculation simulation and molecular docking results

#### 3.4.1 System equilibrium and diffusion behavior

**FIGURE 6:**
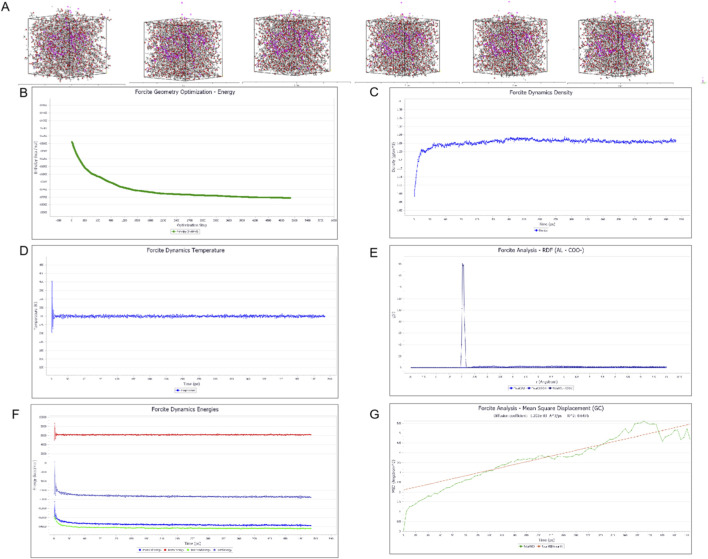
Molecular dynamics (MD) simulation validating system stability and drug transport mechanism. **(A)** Representative snapshots of the molecular system configuration during the simulation trajectory. **(B)** Energy minimization curve demonstrating the convergence to a stable geometric configuration. **(C)** Time-evolution of system density indicating equilibrium under the NPT ensemble. **(D)** Temperature fluctuation profile confirming stable thermal control around 300 K. **(E)** Radial Distribution Function (RDF) analysis highlighting specific coordination interactions between aluminum ions (Al^3+^) and carboxyl groups (COO^−^). **(F)** Thermodynamic stability assessment showing the conservation of total, potential, and non-bonded energies over the simulation trajectory. **(G)** Mean Square Displacement (MSD) of liquiritigenin (LQ) molecules over time; the slope of the linear fit determines the diffusion coefficient **(D)**.

The NPT molecular dynamics simulation of the hydrogel system (“LQ”) was conducted for 500 ps to evaluate its thermodynamic stability and drug transport properties. As shown in Figure 6, the system density stabilized at approximately 1.2 g/cm^3^, and the total energy remained conserved with controlled temperature fluctuations, confirming that the system reached a stable equilibrium suitable for analysis. Crucially, the simulation provided molecular-level insights into the drug release mechanism observed *in vitro*. The Mean Square Displacement (MSD) analysis yielded a diffusion coefficient (D) for liquiritigenin (LQ) of 1.202 times 10^–3^ Å^2^/ps. This relatively low diffusion coefficient indicates that the dense, cross-linked PAA/CMC-Na network significantly restricts the mobility of the drug molecules. This physical parameter correlates directly with the sustained release profile shown in Figure 5G, providing a theoretical basis for the hydrogel’s ability to maintain therapeutic drug concentrations over 24 h without an immediate, uncontrolled burst release. Furthermore, the Radial Distribution Function (RDF) analysis revealed distinct peaks in the interaction probability between Aluminum ions (Al) and Carboxyl groups (COO^−^) within the 0–10.5 Å range. These strong specific interactions confirm the formation of stable coordination bonds acting as physical cross-links. Biologically, this high cross-linking density is essential for maintaining the structural integrity of the hydrogel patch in physiological environments, ensuring that it remains adhered to the wound bed and resists rapid erosion by wound exudates, thereby fulfilling the requirements for a long-lasting wound dressing.

#### 3.4.2 Molecular docking of different target protein molecules of LQ and sepsis

**FIGURE 7:**
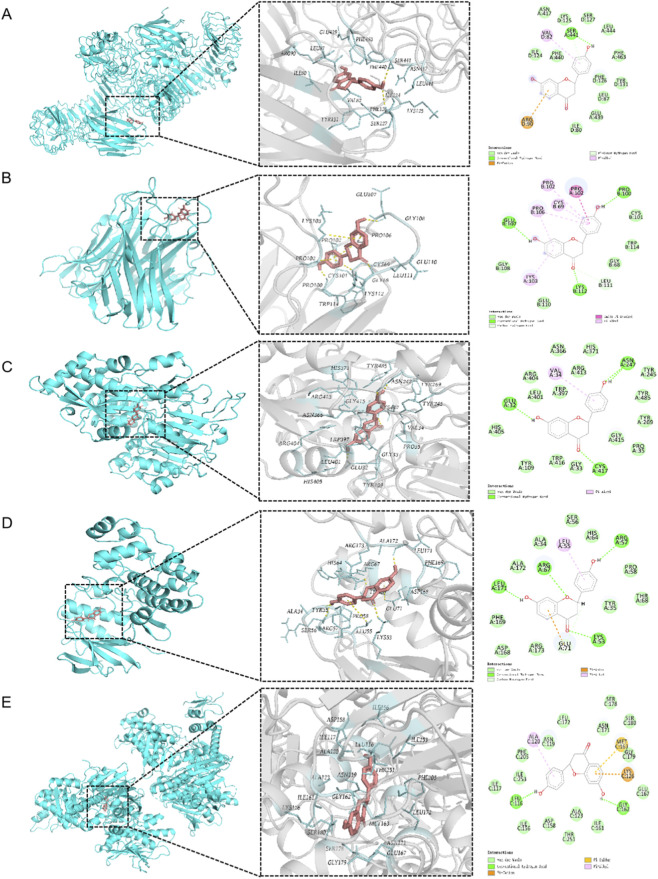
Computational prediction of binding modes between liquiritigenin (LQ) and key sepsis-related target proteins. The panels display the optimal three-dimensional (left) and two-dimensional (right) docking conformations for LQ with **(A)** Toll-like receptor 4 (TLR4), **(B)** Tumor necrosis factor-alpha (TNF-α), **(C)** Plasminogen activator inhibitor-1 (PAI-1), **(D)** p38 mitogen-activated protein kinase (p38 MAPK), and **(E)** High-mobility group box 1 (HMGB1). The 3D visualizations illustrate the ligand occupying the active binding pockets, while the 2D interaction maps detail specific hydrogen bonds, hydrophobic contacts, and electrostatic interactions with key amino acid residues, providing a structural basis for the potential multi-target anti-sepsis mechanism.

Potential therapeutic targets for sepsis, including Toll-like receptor 4 (TLR4), tumor necrosis factor-alpha (TNF-α), plasminogen activator inhibitor-1 (PAI-1), MAPK, and high-mobility group box 1 (HMGB1), were identified from the Protein Data Bank (PDB). Molecular docking of LQ to these target (Figure 7; Table 7) suggests a multi-mechanistic anti-sepsis effect, likely involving TLR4, TNF-α, interleukin-1 β (IL-1β), HMGB1, PAI-1, and p38 MAPK. The highest binding affinity was observed with TLR4 (Score: −7.085 kcal/mol), indicating LQ may act as a potent TLR4 antagonist to competitively inhibit lipopolysaccharide (LPS) binding, thereby blocking the inflammatory signal initiation. Furthermore, LQ displayed favorable affinities for key pro-inflammatory mediators, including TNF-α (−6.434 kcal/mol), IL-1β (−5.586 kcal/mol), and the late-release DAMP, HMGB1 (−5.832 kcal/mol). This suggests LQ can neutralize these mediators or interfere with their receptor interactions, thus suppressing the systemic inflammatory cascade. As a flavonoid, LQ is also proposed to modulate downstream signaling pathways, such as p38 mitogen-activated protein kinase (p38 MAPK), to inhibit the activation of inflammatory transcription factors and reduce mediator synthesis. Finally, LQ’s potential binding to PAI-1 suggests a beneficial role in mitigating sepsis-induced disseminated intravascular coagulation (DIC). Collectively, LQ shows promise as a comprehensive sepsis therapeutic via the synergistic mechanisms of inhibiting inflammation initiation, blocking signal transduction, and neutralizing inflammatory mediators.

**TABLE 7:** liquiritigenin docking scores with potential sepsis target proteins.

Target protein	PDB ID	X	Y	Z	grid size	Glide gscore
TLR4	3FXI	−5	−8	−24	20	−7.085
TNF-α	2TNF	−22	32	−63	20	−6.434
IL-1β	1ILB	10	23	−15	20	−5.586
IL-6	1IL6	4	−2	12	20	−4.055
HMGB1	4Z1I	18	102	20	20	−5.832
Histones H3	1KX5	45	75	8	36	−4.340
GSDMD	6TOS	−45	−20	90	20	−5.059
Thrombomodulin	1BTL	21	0	30	20	−3.985
PAI-1	1B3G	0	25	11	22	−6.388
Rac1	1RTC	0	7	7	20	−4.213
p38 MAPK	1P38	2	21.5	33	23	−5.412

### 3.5 Biological effect evaluation results

**FIGURE 8:**
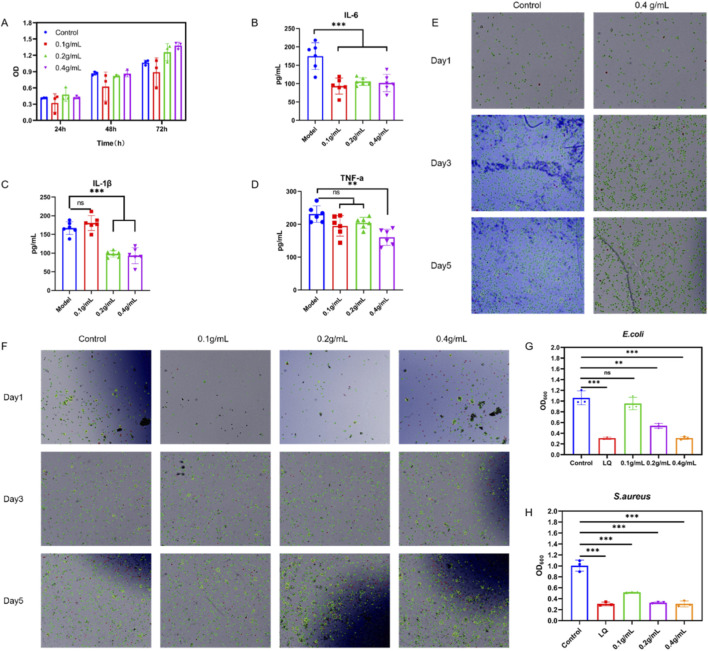
*In vitro* biological evaluation of the hydrogel’s pro-healing, anti-inflammatory, and antimicrobial properties. **(A)** CCK-8 assay assessing the viability of 3T3 fibroblast cells treated with hydrogel extracts at varying time intervals. **(B–D)** Quantitative ELISA analysis of inflammatory cytokines **(B)** IL-6, **(C)** IL-1β, and **(D)** TNF-α levels in LPS-stimulated RAW 264.7 macrophages following treatment with different concentrations of the hydrogel. **(E,F)** Trypan blue exclusion assays monitoring cell proliferation and biocompatibility for **(E)** 3T3 fibroblasts and **(F)** RAW 264.7 macrophages at days 1, 3, and 5. **(G,H)** Antibacterial efficacy demonstrated by OD600 growth curves of **(G)** *E. coli* and **(H)** *S. aureus* in the presence of the hydrogel. Data are presented as mean ± SD. Sample sizes: n = 6 for ELISA experiments and n = 3 for antibacterial and cell viability assays. Statistical significance is indicated by ns p > 0.05, *p < 0.05, **p < 0.01, and ***p < 0.001.

The biological activity of the hydrogel was investigated through *in vitro* assays. Consistent with the cell viability assays, the CCK-8 assay (Figure 8A) confirmed that the extract exhibited no significant cytotoxicity and promoted cell proliferation in a dose-dependent manner. Quantitative analysis revealed that cell viability remained above 98% across all tested concentrations compared to the control group (p > 0.05). This finding was reinforced by Trypan Blue staining (Figure 8E), which demonstrated the significant proliferation of 3T3 fibroblasts over time, with the viability rate consistently maintained above 98%, thereby indicating the material’s biocompatibility and pro-cellular activity. For anti-inflammatory evaluation, an LPS-induced RAW264.7 macrophage inflammation model was employed. ELISA results showed that the release of major inflammatory cytokines—IL-6 (Figure 8B), IL-1β(Figure 8C) and TNF-α(Figure 8D) —was significantly reduced in a dose-dependent manner compared to the LPS-stimulated group. Crucially, concurrent Trypan Blue staining monitoring (Figure 8F) ensured that the observed cytokine reduction was a genuine therapeutic response from healthy, viable cells, excluding any interference or artifact from cytotoxicity. Furthermore, the hydrogel demonstrated a broad-spectrum inhibitory effect against the two primary wound pathogens, the Gram-negative *E. coli* (Figure 8G) and the Gram-positive *S. aureus* (Figure 8H). In summary, this LQ-loaded hydrogel successfully integrates pro-healing, anti-inflammatory, and antimicrobial functionalities, positioning it as an efficient, multifunctional therapeutic platform for complex wound management and the prevention of bacterial infection-induced sepsis.

## 4 Conclusion

This study optimized a multifunctional hydrogel system integrating pro-healing, anti-inflammatory, and antimicrobial capabilities using orthogonal design and response surface methodology. The resulting PAA/CMC-Na matrix demonstrated robust mechanical properties, pH-responsive swelling, and controlled drug release kinetics. Notably, this research integrates Molecular Dynamics (MD) simulations and Molecular Docking analysis to elucidate the drug release mechanism and predict favorable binding modes of LQ with key sepsis targets, including TLR4 and TNF-α. This analysis provided theoretical support for the potential anti-sepsis synergy of LQ.

At the cellular level, the hydrogel demonstrated potential biocompatibility and functional efficacy. The CCK-8 assay confirmed that the hydrogel extract was non-cytotoxic and exhibited a dose-dependent pro-proliferative effect on cells. Trypan Blue staining further supported the material’s biocompatibility, showing that the percentage of viable cells remained consistently above 98%. Its effective anti-inflammatory activity was demonstrated in the LPS-induced RAW264.7 macrophage inflammation model, where ELISA results showed a dose-dependent reduction in the release of inflammatory cytokines TNF-α, IL-6, and IL-1β. Furthermore, the hydrogel displayed a broad-spectrum inhibitory effect against major wound pathogens, including the Gram-negative *E. coli* and the Gram-positive *S. aureus*.

However, it is important to acknowledge the limitations of this study. The current evaluation is restricted to *in vitro* models, which, while promising, cannot fully replicate the complex physiological environment of a living organism. Further comprehensive *in vivo* animal studies and long-term stability assessments are required to fully validate the therapeutic efficacy and safety profile of this system.

Looking ahead, future investigations will prioritize a comprehensive assessment of the hydrogel’s long-term *in vivo* biocompatibility and degradation kinetics to ensure its clinical safety profile. Concurrently, we aim to validate the therapeutic efficacy of this system within specific pathological contexts, with a particular focus on diabetic wound healing models characterized by impaired tissue repair. Furthermore, we will explore the versatility of this hydrogel platform for the co-delivery of synergistic bioactive agents, thereby maximizing its potential to enhance clinical outcomes in complex wound management.

In conclusion, this LQ-loaded hydrogel presents a promising strategy for potentially addressing chronic wound healing issues and preventing bacterial infection-induced sepsis via the sustained local release of the natural compound, LQ.

## Data Availability

The raw data supporting the conclusions of this article will be made available by the authors, without undue reservation.
